# Burden of carbapenem-resistant organisms in the Frankfurt/Main Metropolitan Area in Germany 2012/2013 – first results and experiences after the introduction of legally mandated reporting

**DOI:** 10.1186/1471-2334-14-446

**Published:** 2014-08-19

**Authors:** Niels Kleinkauf, Angelika Hausemann, Volkhard AJ Kempf, René Gottschalk, Ursel Heudorf

**Affiliations:** Public Health Authority of Frankfurt/Main, Breite Gasse 28, D- 60313 Frankfurt, Germany; University Hospital, Johann Wolfgang Goethe-University, Frankfurt/Main, Germany

**Keywords:** Gram-negative bacteria, Multidrug resistance, Carbapenemase, Mandatory reporting, Epidemiology, Population-based surveillance

## Abstract

**Background:**

The federal state of Hesse, Germany, introduced a laboratory-based reporting scheme for carbapenem-resistant organisms (CROs).

**Method:**

The results of the first year of mandated reporting of CROs from April 2012 through March 2013 to the Public Health Authority of Frankfurt/Main, responsible for a population of 700,000 inhabitants, are described.

**Results:**

Within a period of 12 months 243 CROs were notified to the health authority. Of these 213 isolates had been reported from 16 of the 17 hospitals in Frankfurt/Main, 6 from ambulatory settings and 24 from clinics outside of Frankfurt/Main. Mean incidence rate per 1,000 patient days in hospitals was 0.138 (range 0.02-0.28).

**Conclusion:**

In Frankfurt/Main almost all hospitals have reported CROs in the study period though the frequency of isolation varies strongly and many facilities only report CROs sporadically. Molecular data indicate a high diversity of different carbapenemases. Autochthonous transmission must be assumed despite the absence of major outbreaks. Rapid and coordinated efforts by clinicians and health departments are crucial to control the spread of CRO infections. The mandatory reporting scheme provides important data to guide the implementation of preventive measures.

**Electronic supplementary material:**

The online version of this article (doi:10.1186/1471-2334-14-446) contains supplementary material, which is available to authorized users.

## Background

For nearly 20 years carbapenems had retained almost universal activity against *Enterobacteriaceae* [1], but in recent years resistance is accumulating globally. The current increased use of carbapenems is a consequence of the growing prevalence of cephalosporin-resistant pathogens. This cycle of increased antimicrobial usage and escalating resistance leads to an inevitable tension between the good of the individual patient and that of public health. Furthermore the applicability of this indispensable group of second-line antibiotics is jeopardized.

Increasing prevalence of carbapenem-resistant organisms (CROs) in healthcare facilities has been reported not only from Asia, but also from the USA and Europe [2–5]. First reports of outbreaks due to carbapenem-resistant *Enterobacteriaceae* (CRE) in Germany, particularly those producing *Klebsiella pneumoniae* carbapenemase (KPC) have been published [6, 7] in 2009. Carbapenem-resistance can be conferred by the presence of various mechanisms of resistance. The currently most significant development is the world-wide spread of carbapenemases, beta-lactamases capable of hydrolysing most beta-lactams, including carbapenems. Carbapenemases such as OXA-48, KPC- and VIM-type carbapenemases are the most prevalent in Germany [8]. Furthermore, the number of NDM-type carbapenemases are increasing slowly, with recent reports of travel-related cases, autochthonous cases and outbreaks [9, 10].

Whereas 13 European countries have included carbapenem-resistant organisms (CROs) in their list of mandatorily notifiable entities in recent years, no reporting obligation has been introduced at national level in Germany to date [11]. Federal states in Germany, however, may mandate reporting of specified conditions by local state law. Hesse, one of the federal states of Germany, was the first federal state to introduce a mandatory reporting scheme for carbapenem-resistant gram-negative pathogens in November 2011 [12]. Detailed reporting criteria (Table 1) were issued in a separate decree which came into effect in April 2012.

**Table 1:** **Reporting criteria for gram-negative bacteria, Hesse, Germany**

**1.**	**Every detection of a carbapenemase by molecular methodology** ^**1**^
**2.**	**Phenotypic resistance tests (culture + antibiogram)**
	**a)** ***Pseudomonas aeruginosa***
	If resistant to all three of the following antibiotics:
	Imipenem, meropenem and ceftazidime
	**b)** ***Enterobacteriaceae*** **(if not mentioned elsewhere)**
	If resistant to at least one of the following antibiotics:
	Imipenem, meropenem or ertapenem
	**c)** ***Proteus spp.***
	***Morganella spp.***
	***Providencia spp.***
	***Serratia spp*** *.*
	In case of resistance to imipenem *and* resistance to ertapenem *or* meropenem (an isolated increase of the MIC for imipenem together with normal MICs for meropenem and – if performed – for ertapenem does not require reporting)
	**d)** ***Acinetobacter baumannii-complex***
	***Enterobacter spp.***
	***Citrobacter spp.***
In case of resistance to imipenem *or* meropenem. (isolated resistance to ertapenem does not require reporting)	
Not to be reported are species with intrinsic carbapenem-resistance. These include:	
***Stenotrophomonas maltophilia***	
***Elizabethkingia meningoseptica***	
***Chryseobacterium indologenes***	
***Burkholderia cepacia***	

^1^Laboratories are encouraged but not obliged to type carbapenem-resistant bacteria, analyses are offered free of charge by the national reference centre.

Here we present the results of the first year of mandatory reporting of CROs in Frankfurt/Main and the Rhine-Main Region.

## Methods

The introduction of the new reporting scheme obliged laboratories in the federal state of Hesse to report by fax every detection of a carbapenem-resistant gram-negative organism (according to the criteria listed in Table 1) within 24 hours directly to the local health authority in charge. The results presented in this report include all notifications received by the public health authority of Frankfurt/Main from April 2012 (the introduction of detailed reporting criteria) through March 2013. These include notifications from all patients treated in Frankfurt’s health care facilities as well as notifications of the city’s residents treated in health care facilities outside of the city of Frankfurt/Main. Frankfurt/Main represents a typical city at the heart of a large metropolitan area concentrating highly specialized health care facilities within the region. By number Frankfurt holds 10% of the hospitals in the federal state of Hesse (17/172), yet these include a number of large tertiary care hospitals whose catchment areas reach far beyond the city’s boundaries. The city’s 700,000 inhabitants represent 12% of the federal states population (6.1 million). Its hospitals provide 6,240 beds (17.2% of beds provided by the state) or 1,594,388 bed days (15.7%) compared to 36,229 beds or 10,179,034 bed days state wide. Published results of the mandated reporting of CRO’s in Hesse display a total of 549 notifications between January 2012 and April 2013 at state level compared to 252 (23.1%) notifications (these include 219 notifications from Frankfurt/Main in the study period of this publication + 33 notifications from Jan 2012 through Mar 2012) for Frankfurt/Main [13].

Upon receipt lab reports were reviewed by the health authority. Additional clinical information was requested from the treating physician. On identification of possible clusters individual cases were followed up by contacting the hygiene staff of the concerned facilities by phone. If necessary infection control measures were recommended.

Reports were saved in an Excel file and included the following information: ID; name of patient, date of birth, sex, place of residence, date of hospitalization, infection/colonisation status, sampling date, previous hospitalizations and travel history, isolation precautions, genus and species of the detected organism, susceptibility profile and if applicable also the result of carbapenemase testing. Calculations were performed with SPSS version 15 software.

The number of total patient days required for the calculation of the incidence was provided on a quarterly basis by the participating hospitals.

Every notification of a pathogen not previously reported for the patient was counted as a case. Since more than one resistant pathogen was identified for several patients, the number of cases exceeded the number of patients.

Ethical approval was not required for this descriptive study as the information presented is based exclusively on anonymized data from legally mandated public health surveillance and contains no direct or indirect person identifiers.

## Results

**Figure 1:**
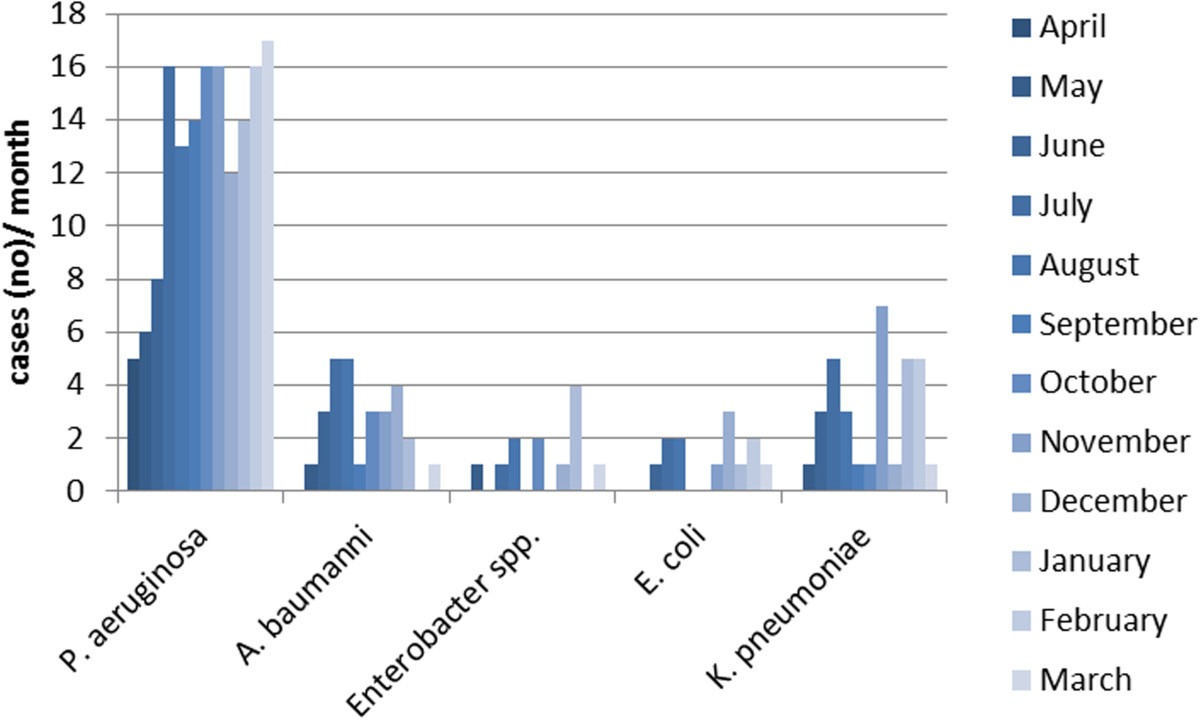
**Notifications of CROs – Frankfurt/Main, 1.4.2012-31.3.2013.**

From April 2012 through March 2013 the Public Health Authority of Frankfurt/Main received reports of 243 multidrug-resistant gram-negative organisms identified according to the reporting criteria from 225 patients: these included 30 isolates in the second quarter of 2012, 70 in the third, 73 in the fourth quarter of 2012 and 70 in the first quarter of 2013 (Figure 1). *P. aeruginosa* was the most frequently notified species (153), followed by *K. pneumoniae* (33), *A. baumannii* (28), *E. coli* (13), *Enterobacter spp.* (12) and other *enterobacteriaceae* (4) with carbapenem-resistance. The identification of a carbapenemase was reported for 32 pathogens (Table 2). The total number of tests performed to identify carbapenemases is not known.

**Table 2:** **Demographic characteristics and history of hospitalization abroad of patients colonized or infected with CRO, Frankfurt/Main, 1.4.2012- 31.03.2013**

	Total	*P. aeruginosa*	*A. baumannii*	*K. pneumoniae*	*E. coli*	*Enterobacter*
**Total**	243^a^	153	28	33	13	12
**Age (years) (mean)**	58.9 ± 20.1	59.5 ± 19.7	53.1 ± 17.2	59.4 ± 19.5	59.2 ± 23.3	58,0 ± 30,7
**Age range (years)**	0-97	0-97	18-84	0-90	2-91	0-89
**Percentage of male patients**	66.7	60.1	82.1	81.8	61.5	83.3
**Hospitalization abroad during previous 6 months**	32 patients^b^	N = 10	N = 11	N = 7	N = 2	N = 1
		Iran	Egypt	Egypt	Qatar	Sri Lanka
		Kuwait	Brasil	Greece	India	
		Brasil	Ukraine	Nicaragua		
		Qatar	Qatar	Italy		
		India	Kosovo	India (n = 2)		
		Egypt	Russia	Turkey		
		Nicaragua	India			
		Venezuela	Thailand			
		Russia	(n = 2)			
		Macedonia	Turkey			
			Morocco			
**Place of residence abroad**	N = 21 (8.6%)	N = 11	N = 6	N = 2	N = 2	
		Egypt	Algeria	Egypt	India	
		Iran	Egypt	India	Egypt	
		India	Qatar			
		Kuwait	Kuwait			
		Macedonia	Russia (n = 2)			
		Qatar (n = 2)				
		Russia				
		Saudi Arabia				
		Ukraine				
		Venezuela				
**Infection (%)**	29.7	24.2	28.6	30.3	23.1	50.0
**Carbapenemase (cases)**	32 cases^c^	*Metallo-ß-lactamase, unspecified (n = 3)*	*OXA-23-like (n = 7)*	*OXA-23-like (n = 1)*	*OXA-48 (n = 4)*	
		*VIM-1 (n = 1)*	*NDM-1 (n = 1)*	*OXA-48 (n = 4)*	*NDM-7 (n = 1)*	
		*VIM-2 (n = 3)*		*NDM, not further specified (n = 3)*		
				*KPC-2 (n = 1)*		
				*KPC-3 (n = 1)*		

^a^including *C. koseri*, *E. sakazakii*. 1 *K. oxytoca*, *P mirabilis.*

^b^including 1 patient, *C. koseri* Libya.

^c^including OXA-48 (n = 1) from *C. koseri*; NDM1/6 (n = 1) from *K. oxytoca*.

The Public Health Authority received notifications for 213 CROs from 16 of the 17 hospitals in Frankfurt/Main, 6 reports were from outpatient facilities in Frankfurt/Main and 24 notifications came from hospitals outside of city, for patients under the responsibility of the Frankfurt City Public Health Authority, due to their registered place of residence. While 48% of the notifications originated from a single tertiary care hospital (103 notifications, 0.28/1,000 patient days), the other hospitals notified 22 (0.20/1,000 patient days), 18 (0.19/1,000 patient days), 14 (0.09/1,000 patient days), two hospitals notified 13 (0.13 and 0.06/1,000 patient days), 1 × 8 (0.15/1,000 patient days), 1 × 6 (0.11/1,000 patient days), 1 × 4 (0.04/1,000 patient days), 1 × 3 (0.05/1,000 patient days), 3 × 2 (0.03, 0.05, 0.05/1,000 patient days), 3 × 1 isolates (0.02, 0.02, 0.05/1,000 patient days) respectively. The absolute frequency given above does not reflect the size and occupancy of the hospital and is better expressed by the yearly incidence/1,000 patient days (given in parentheses). The average yearly incidence was 0.138 notifications/1,000 patient days. 67% of the notifications concerned isolates from male patients. Regarding the patients from outpatient facilities, all patients had their place of residence in Germany, 5 in Frankfurt/Main, 1 in a neighbouring district in Hesse, none reported prior hospitalization, 1 reported previous travel abroad (Serbia), 5 were colonized 1 infected with a CRO and 3 were carriers of an identified carbapenemase (VIM-2, OXA-23-like carbapenemase, unspecified metallo-carbapenemase). The average age of the patients was 60 ± 20 years ranging from 0–97 years. 45% of the notified patients had their main residence in Frankfurt/Main, 28% were residents of the Rhine-Main region (bordering to the city of Frankfurt/Main) and a further 18% were German residents outside of the Rhine-Main region. 9% of patients had their place of residence abroad. Thirty-two patients (13% of all patients) had received healthcare in a foreign country (Table 2).

Two-thirds of the isolated pathogens were reported as colonizers, 30% as causative agent of infections. One third (34%) of all isolates originated from sputum or tracheal aspirates, 23% from wound swabs, 18% from skin or perineal swabs, 17% from urine samples, 3% from blood cultures and 5% from other materials.

**Figure 2:**
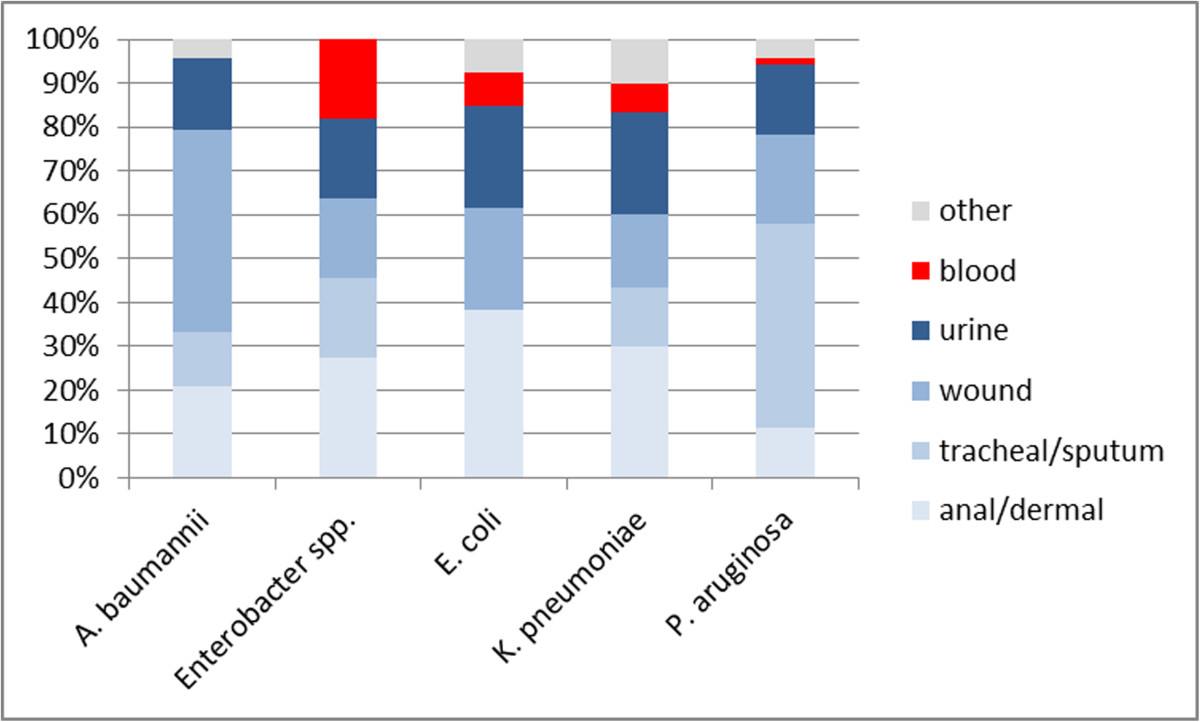
**Notified CROs by species and sampling site, Frankfurt/Main, 1.3.2012-31.4.2013.**

No significant differences were observed with regard to age, sex and history of previous hospitalization between different species. However, the sampling sites from which the different pathogens were isolated were highly diverse. *P. aeruginosa* was isolated most frequently from tracheal aspirates (46%), *A. baumannii* from wound swabs (46%) and *Enterobacter spp.* from skin or perineal swabs (27%) (Figure 2).

Twenty-six per cent of the samples had been taken within the first two days of hospitalization and 22% of the CROs had been detected after having been hospitalized for more than a month.

## Discussion

Carbapenem-resistant pathogens are an increasing threat world-wide, in Europe [14] and also in Germany [8, 15]. In 2010 Grundmann *et al.* proposed a five-level staging system to index the magnitude of carbapenem-non-susceptibility across Europe. Germany was categorized into level 3 indicating regional spread together with Hungary and France. Only Italy, Poland (level 4), Greece and Israel (level 5) were categorized into higher levels [16]. According to this staging scheme, a group of European experts reassessed the situation in 2013. Germany remained in the category 3 - together with France, Croatia, Kosovo, United Kingdom, Spain, and Poland, whereas meanwhile three countries (Hungary, Israel, and Ireland) had been categorized in stage 4, and three other countries (Greece, Italy and Malta) in category 5 [11].

In Frankfurt/Main CROs had already been reported sporadically and sent to reference laboratories in previous years on the basis of a general clause in the German Protection against Infection Act requiring notification of any cluster of nosocomial infections which may constitute an outbreak of public health relevance. The first notifications of CROs to the Frankfurt City Public Health Authority were received in the years 2005 to 2007 primarily reporting outbreaks with *A. baumannii* in Frankfurt hospitals [17]. In 2007 an *A. baumannii* producing the New Delhi metallo-β-lactamase-1 (NDM-1) had been isolated for the first time in Frankfurt/Main from a patient who had been repatriated to Germany from Serbia [18, 19]. With the obligation to report carbapenem-resistant organisms in the federal state of Hesse, the availability and quality of data have improved considerably, particularly as all laboratories are obliged to follow these regulations, including hospital-based and outpatient laboratory facilities. The detection of these organisms is however influenced by the quality and frequency of clinical microbiological analyses and screening schemes, as well as by case-mix of the medical institutions in which the samples have been drawn. Therefore, an interpretation based on the number of patients or on incidence rates must be done carefully, and the possibility of inter-facility comparison is limited.

Within the first year of mandatory reporting the Frankfurt City Public Health Authority received 243 notifications of CROs. Carbapenem-resistant gram-negative organisms were reported from nearly all inpatient facilities and also a few outpatient facilities in Frankfurt/Main. Thus, carbapenem-resistance is not solely a problem confined to a few highly specialized hospitals. The problem has meanwhile reached all healthcare facilities, even though cases are distributed disproportionately and 48% of all notifications were received from a single hospital which however reported only 23% of the total of patient days registered for Frankfurt’s healthcare facilities. A possible explanation for such high incidences in some of the facilities is that these are large tertiary care clinics some of which treat a relatively high proportion of patients in intensive care units and other large highly specialized treatment units. Many of the patients treated here are immunocompromised and highly susceptible to bacterial infections. The widespread presence of CROs in Frankfurt’s hospitals points towards autochthonous transmissions in the region. Yet large inter-institutional outbreaks have not been reported and similarly investigations performed by the local health authority gave no indication of such events. None of the patients treated in the outpatient facilities reported a history of prior hospitalization. This may be an indication of ongoing community transmission. Though the number of reported carbapenemases may not reflect their true prevalence, the results of those identified and reported indicate a high diversity of different carbapenemases in the region (Table 2).

30% of the notifications reflected infections with carbapenem-resistant organisms, of which 31% were wound-, 23% urinary tract-, 19% respiratory tract-infections and 11% bloodstream infections. 71% of the patients with CROs had a history of hospitalization within the previous 6 months indicating that prior hospitalization can serve as a risk factor for the carriage of CROs. Furthermore 21 of these notifications (8.6%) related to patients who had previously been treated in hospitals abroad, in countries or regions with a known high prevalence of CROs. Several of the patients had been transferred directly from hospitals abroad for specialised treatment into medical facilities in Frankfurt/Main. This may be an indication of a substantial cross-border transfer of medical patients.

26% of the notifications were related to patients who had been hospitalized less than 48 hours. The occurrence of CROs in patients who have only recently been admitted to the hospitals may be an indication of an acquisition of the pathogen in the community setting or by undiagnosed acquisition in another previously visited health care institution.

For several of the patients reported here, the carrier status for CRO was known on admission so that these patients were isolated pre-emptively, as suggested in the latest recommendations of the German Commission for Hospital Hygiene and Infectious Disease Prevention published in October 2012 [20]. The other patients with carbapenem-resistant organisms were isolated only after laboratory findings had been communicated and a few of these patients had already been dismissed when the information became available, so that a transfer of the CROs to further patients could not be excluded with certainty. In two cases for which a transmission of the pathogen was suspected, extensive contact tracing and environmental investigations were performed, ending with negative results. These patients were colonized with *Klebsiella spp.* both carrying an NDM-type carbapenemase.

The CDC guidance for control of carbapenem-resistant *enterobacteriaceae* (CRE) recommends different prevention strategies, depending on the prevalence of CRE within a region [21]. Regions with few CRE identified are defined as those where the majority of healthcare facilities do not regularly have patients with CRE admitted. This includes regions where some facilities may have several CRE colonized or infected patients but are surrounded by facilities with only a few (e.g. less than one per month) or none. This situation is applicable to Frankfurt/Main in 2012/2013.

CDC recommends aggressive measures to control the problem in regions with few CRE identified by making CRE a reportable event, closely monitoring the situation including periodically surveying acute and long-term care facilities for the presence of CRE and providing feedback of the results of the data analysis to the facilities. An important aspect is the identification of facilities with high CRE prevalence and communication of this information to other facilities. Furthermore, health departments should cooperate closely with infection prevention personnel, monitor implementation of preventive measures in facilities with CRE and provide training and timely information to facilities that have so far not identified CRE patients. Finally the inter-facility communication of CRE findings needs to be encouraged.

Some of these aspects have already been implemented in Hesse and the Rhine-Main region. CROs have been made a reportable event in the federal state of Hesse. The Hessian Hygiene Act 2011 [22] explicitly requires healthcare facilities which transfer patients to other facilities to inform these about possible CRE findings. However, to date this has not been consistently implemented. A local network of medical and nursing professionals (MRE-Net Rhine-Main) has offered several trainings in 2012 covering the topic of multidrug-resistant gram-negative pathogens following the publication of the latest recommendations of the German Commission for Hospital Hygiene and Infectious Disease Prevention. Further work is necessary to improve the inter-facility communication on carriage of multidrug-resistant pathogens.

Data of current regional surveys are available. In May 2012 > 1,000 ambulatory dialysis patients were screened for multidrug-resistant pathogens by two cooperating local professional networks (MRSAar-Net and MRE-Net Rhine-Main) and these included 751 patients from the Rhine-Main region. Forty of these patients (7.5%) were colonized with ESBL but all were carbapenem-sensitive. In November 2012, however, the first two patients with CROs from an outpatient dialysis facility in Frankfurt were identified that had been notified according to the new mandate to report CROs in Hesse. Both were colonized with a carbapenem-resistant *K. pneumoniae*, one of which carried an OXA-23-like carbapenemase. In autumn 2012 a total of 150 residents out of 8 of 40 nursing homes for the elderly in Frankfurt/Main were screened for multidrug-resistant pathogens. ESBL were identified in 40 (27%) of the residents, however, no isolate exhibited resistance towards carbapenems.

The new mandate appears to have been rapidly accepted and implemented successfully within the first four months (April-July) by laboratories and other health care facilities in Frankfurt/Main as judged by the number of notifications which appear to have stabilized after a first sharp increase and by the predominantly positive feedback received. A meaningful comparison of the data will however only be possible after the data have been collected over a longer period allowing the identification of trends in time.

There are several limitations to this descriptive study of reporting data. As underreporting is a typical feature of passive surveillance systems the reported case numbers only represent the lowest possible spread of CROs in the region. Particularly in the beginning of the study an increase of the reported number of cases could be observed which most probably corresponds to a brief period of familiarization with the new reporting obligation rather than a true increase in the number of cases. Furthermore the detail of information which laboratories and clinicians can be obliged to report will always be limited within the framework of what can reasonably be expected in their routine workflow. Details of laboratory methodology (antibiotic susceptibility testing standards, selection criteria for typing, typing methodology, phenotypic/genotypic test systems, different sensitivity rates) have not been documented and may vary between different laboratories. Finally several factors influencing the frequency of detection of CROs could not be asked for such as details on the established screening programs and the criteria applied in different hospitals, risk factors for the carriage of CROs such as prior antibiotic treatment and organizational differences between hospitals including differences in case mix and specialization of units which may contribute to the differences in the incidence of CROs between the hospitals.

All results from the reporting system, as well as those from the surveys performed are shared with the healthcare facilities of Frankfurt/Main. Major efforts including information events and trainings are being made in close cooperation with the healthcare facilities aiming to fulfil the hygiene standards and to ensure that antibiotics are used appropriately in order to minimize the spread of CROs as effectively as possible. Adequacy and compliance with established hygiene procedures and documentation and assessment of CRO findings are evaluated regularly in the facilities by the health authority. In view of the wide-spread distribution of these pathogens and the increasing cross-border transfer of patients, however, a regional approach alone will not be sufficient.

## Conclusions

In Frankfurt/Main most healthcare facilities have reported CROs in the study period. While a few facilities identified CRO colonized or infected patients regularly the majority of facilities still encounter CROs on an infrequent basis. Although dissemination of CROs mainly occurs among hospitalized patients there are also indications of cases in ambulatory settings which could be a sign of ongoing community transmission. Regarding 243 CROs in twelve months in the Frankfurt/Main metropolitan area a well-concerted effort is needed to prevent the further spread of CROs in order to avoid an uncontrollable situation as can now be observed worldwide for ESBL producers. Laboratory capacities for molecular testing need to be strengthened. The data gathered by legally mandated reporting of CROs are needed to guide infection–control measures taken to limit the further spread of these antibiotic-resistant pathogens. The introduction of a countrywide mandate for reporting would allow to recognise regional differences and to focus prevention efforts accordingly.

## Authors’ original submitted files for images

Below are the links to the authors’ original submitted files for images. Authors’ original file for figure 1 Authors’ original file for figure 2 Authors’ original file for figure 3

## Abbreviations

CDC: Centers for Disease Control and Prevention, Atlanta, USA
CRO: Carbapenem-resistant organism
CRE: Carbapenem-resistant Enterobacteriaceae
ESBL: Extended-spectrum ß-lactamase
MRE–Net Rhine: Main - Regional network of health authorities and healthcare facilities to coordinate infection tracking and control efforts for MRSA and other multidrug-resistant organisms, Hesse, Germany
MRSAar-Net: Regional network, Saarland, Germany
MRSA: Methicillin-resistant *Staphylococcus aureus*
MDRO: Multidrug-resistant organism.
