# Supplementary figures and images for: Burden of carbapenem-resistant organisms in the Frankfurt/Main Metropolitan Area in Germany 2012/2013 – first results and experiences after the introduction of legally mandated reporting
Source: BMC Infect Dis. 2014 Aug 19;14:446. doi: 10.1186/1471-2334-14-446 (PMC4143562; doi:10.1186/1471-2334-14-446)

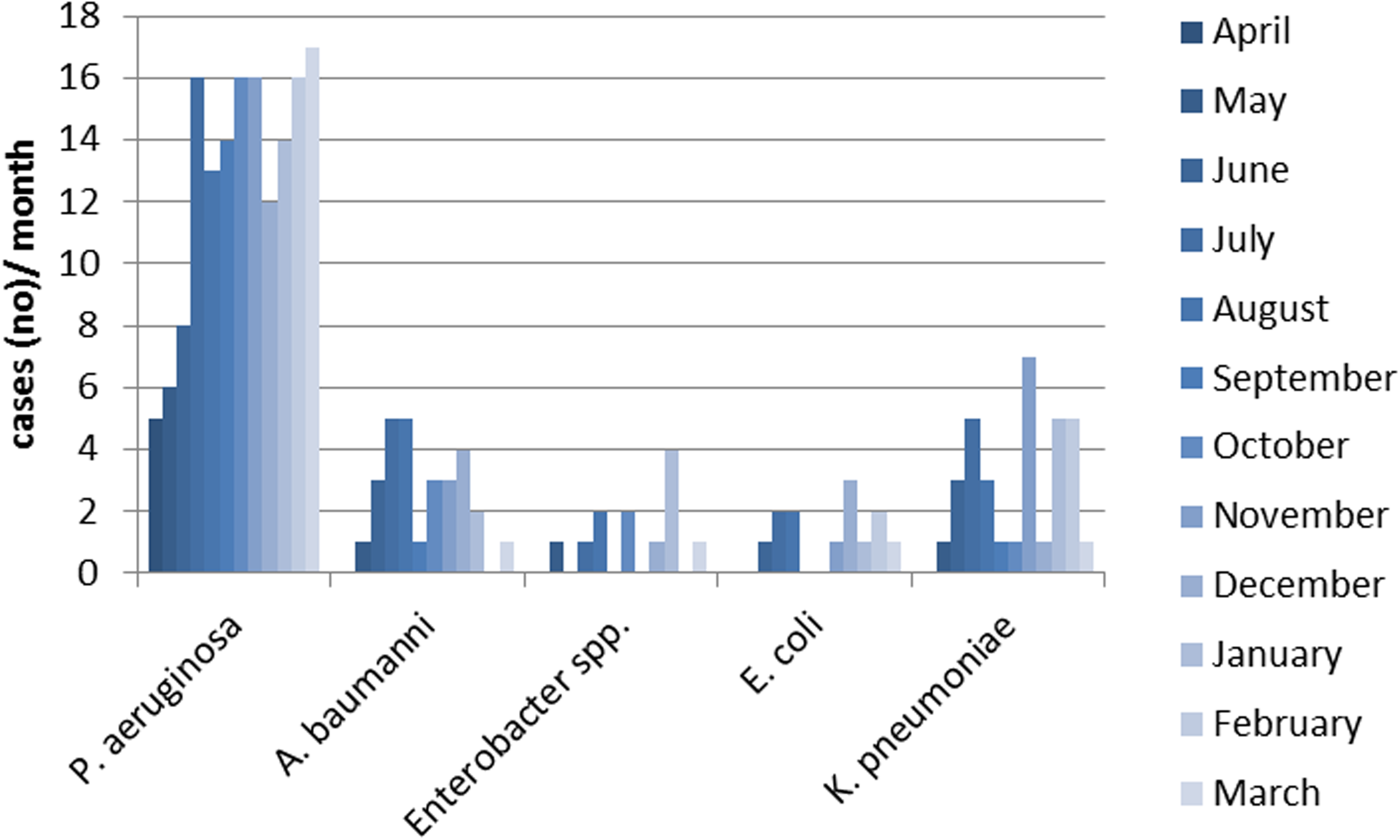

Supplement: Supplementary file 1 — Authors’ original file for figure 1 [file 12879_2014_3743_MOESM1_ESM.tif]

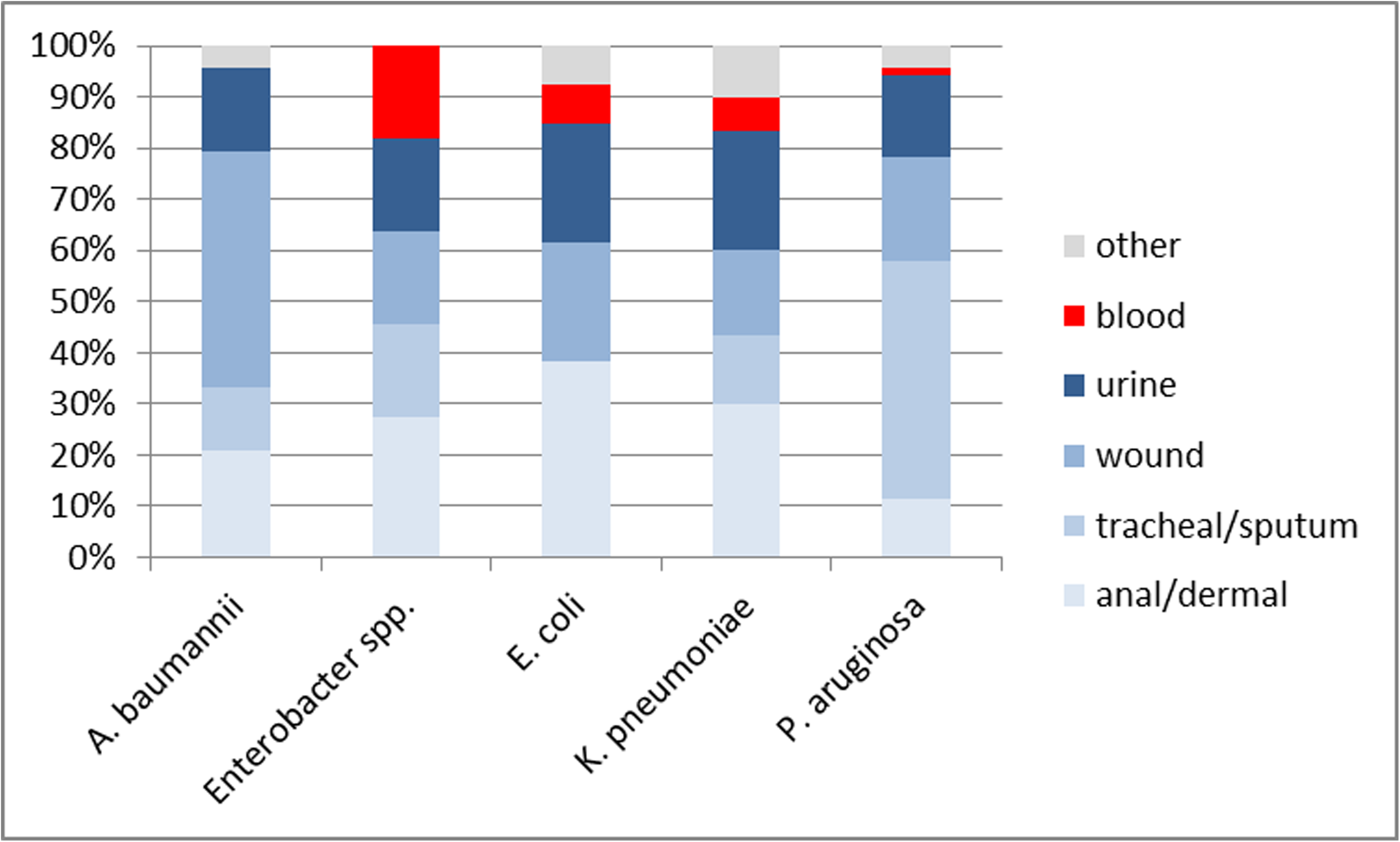

Supplement: Supplementary file 2 — Authors’ original file for figure 2 [file 12879_2014_3743_MOESM2_ESM.tif]

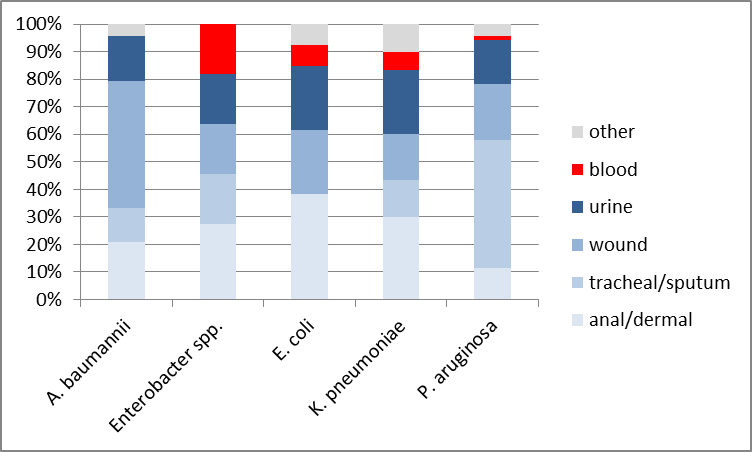

Supplement: Supplementary file 3 — Authors’ original file for figure 3 [file 12879_2014_3743_MOESM3_ESM.png]
